# Supplementary material for: The Effect of Employee-Oriented Flexible Work on Mental Health: A Systematic Review
Source: Healthcare (Basel). 2022 May 10;10(5):883. doi: 10.3390/healthcare10050883 (PMC9141970; doi:10.3390/healthcare10050883)
Supplement: Supplementary file 1 [file healthcare-10-00883-s001.zip › healthcare-1695599-supplementary.pdf]

healthcare

MDPI

Supplementary Tables

Table S1. Literature searches made in October-November 2020**.**

Query	Filters	Results
**PubMed 5.11.2020**		
(work schedule tolerance[Mesh] OR shift work schedule[Mesh] OR workplace[Mesh] OR workplace[tiab] OR flexitime[tiab] OR work shift[tiab] OR work schedule[tiab] OR flexible work[tiab] OR flexiwork[tiab] OR flexi work[tiab] OR flexi work arrangement[tiab] OR flexible working arrangement[tiab] OR shift work [tiab] OR time management[Mesh] OR time management[tiab] OR workplace[Mesh] OR workplace[tiab] OR flexible work arrangements[tiab] OR telecommuting[tiab] OR return to work[Mesh] OR return to work[tiab] OR working time[tiab] OR work life conciliation[tiab] OR work-life conciliation[tiab] time management[Mesh] OR time management[tiab] OR telework[tiab] OR working hours[tiab] OR schedule control[tiab] OR remote work[tiab] OR work family interference[tiab] OR work family conflict[tiab] OR work family interface[tiab] OR part-time work[tiab] OR work-life conflict[tiab] OR work life conflict[tiab] OR work/family[tiab] OR remote work[tiab] OR care arrangements[tiab] OR career break[tiab] OR job sharing[tiab] OR term-time work[tiab] OR compressed working time[tiab] OR self-rostering[tiab] OR sabbatical[tiab] OR work-life reconciliation[tiab] OR work life reconciliation [tiab]) AND (mental disorders[Mesh] OR mental disorders[tiab] OR depression[Mesh] OR depressive disorder[Mesh] OR depress*[tiab] OR anxiety[Mesh] OR anxiety disorders[Mesh] OR anxiety[tiab] OR burnout, psychological[Mesh] OR exhaustion, psychological[Mesh] OR exhaustion[Mesh] OR exhaustion[tiab] OR burnout, professional[Mesh] OR burnout[tiab] OR affective disorders, psychotic[Mesh] OR mood disorders[Mesh] OR mood disorders[tiab] OR antidepressive agents[Mesh] OR antidepressive agents, tricyclic[Mesh] OR antidepressants[Mesh] OR monoamine oxidase inhibitors[Mesh] OR serotonin and noradrenaline reuptake inhibitors[Mesh] OR psychotropic drugs[Mesh] OR citalopram[Mesh] OR fluvoxamine[Mesh] OR antimanic agents[Mesh] OR antidepressive agents, second-generation[Mesh] OR psychiatric distress[tiab] OR work engagement[tiab] OR mental health[tiab] OR mental wellbeing[tiab] OR mental illness [mesh] OR mental illness [tiab] OR psychiatric disorders[mesh] OR psychiatric disorders[tiab] OR psychiatric illness [mesh] OR psychiatric illness[tiab])	English	1,915
**Scopus 31.10.2020**		

Query	Filters	Results
((workplace* OR flexitime* OR "work* shift*" OR "work* schedule*" OR "flexible* work*" OR flexiwork* OR "flexi* work*" OR "shift* work*" OR "time* management*" OR telecommuting* OR "return* to work*" OR "work* time*" OR "work* life* conciliat*" OR telework* OR "work* hour*" OR "schedule* control*" OR "remote* work*" OR "part-time* work*" OR "work-life* conflict*" OR "work* life* conflict*" OR "work* family*" OR "care* arrangement*" OR "career* break*" OR "job* shar*" OR "term-time* work*" OR "compres* work* time*" OR "self* roster*" OR sabbatic* OR "work* life* reconc*" OR "work-life* reconc*")) AND (TITLE-ABS-KEY ("mental* disorder*" OR depress* OR anxiety* OR burnout* OR "burn-out*" OR exhaustion* OR "affective* disorder*" OR "mood* disorder*" OR "antidepressive* agent*" OR antidepressant* OR "monoamine* oxidase* inhibitor*" OR "serotonin* and noradrenalin* reuptake* inhibitor*" OR psychotropic* OR citalopram* OR fluvoxamin* OR "antimanic* agent*" OR "antidepressive* agent*" OR "psychiatric* distress*" OR "work* engagement*" OR "mental* health*" OR "mental* wellbeing*" OR "mental* well-being*" OR "mental* illness*" OR "psychiatric* disorder*" OR "psychiatric* illness*")) AND (LIMIT-TO (LANGUAGE , "English"))	English	3,997
**Web of Science 31.10.2020**		
(workplace* OR flexitime* OR "work* shift*" OR "work* schedule*" OR "flexible* work*" OR flexiwork* OR "flexi* work*" OR "shift* work*" OR "time* management*" OR telecommuting* OR "return* to work*" OR "work* time*" OR "work* life* conciliat*" OR telework* OR "work* hour*" OR "schedule* control*" OR "remote* work*" OR "part-time* work*" OR "work-life* conflict*" OR "work* life* conflict*" OR "work* family*" OR "care* arrangement*" OR "career* break*" OR "job* shar*" OR "term-time* work*" OR "compres* work* time*" OR "self* roster*" OR sabbatic* OR "work* life* reconc*" OR "work-life* reconc*") Timespan: 1945-2020. Indexes: SCI-EXPANDED, SSCI, A&HCI, CPCI-S, CPCI-SSH, BKCI-S, BKCI-SSH, ESCI.	# 1	41,613
TS=("mental* disorder*" OR depress* OR anxiety* OR burnout* OR "burn-out*" OR exhaustion* OR "affective* disorder*" OR "mood* disorder*" OR "antidepressive* agent*" OR antidepressant* OR "monoamine* oxidase* inhibitor*" OR "serotonin* and noradrenalin* reuptake* inhibitor*" OR psychotropic* OR citalopram* OR fluvoxamin* OR "antimanic* agent*" OR "antidepressive* agent*" OR "psychiatric* distress*" OR "work* engagement*" OR "mental* health*" OR "mental* wellbeing*" OR "mental* well-being*" OR "mental* illness*" OR "psychiatric* disorder*" OR "psychiatric* illness*") Timespan: 1945-2020. Indexes: SCI-EXPANDED, SSCI, A&HCI, CPCI-S, CPCI-SSH, BKCI-S, BKCI-SSH, ESCI.	#2	1,128,614
#2 AND #1	#3	3,933
#2 AND #1	#4	3,767

Query	Filters	Results
Refined by: LANGUAGES: (ENGLISH)		
**PsycINFO 30.11.2020**		
workplace*.ti.	#1	10678
work*.ti.	#2	133895
job*.ti. (23601)	#3	23601
exp *Occupations/ or (employee* or employed* or employer*).ti.	#4	63449
(staff* or (organization* or organisation*)).ti.	#5	66025
or/1-5	#6	251735
exp Work Scheduling/ or work* schedul*.mp.	#7	3762
exp Workday Shifts/ or shift* work*.mp.	#8	3243
flexitime*.mp.	#9	52
work* shift*.mp.	#10	2498
flexi* work*.mp.	#11	1083
flexiwork*.mp.	#12	4
exp Time Management/ or time* manag*.mp.	#13	5467
exp Telecommuting/ or telecommut*.mp.	#14	498
exp Reemployment/ or return* to work*.mp.	#15	3739
work* time*.mp.	#16	1590
work* life* conciliat*.mp.	#17	3
telework*.mp.	#18	356
work* hour*.mp.	#19	3388
schedul* control*.mp.	#20	595
(remote* adj1 work*).mp.	#21	202
care* arrangement*.mp. or exp Child Care/	#22	11567
career* break*.mp. or exp Occupational Choice/	#23	8929
((job* or work*) adj1 shar*).mp.	#24	562
compres* work* time*.mp.	#25	1
self* roster*.mp.	#26	5
sabbatical*.mp.	#27	124
((part* time* or term* time*) adj2 (work* or job* or emplo*)).mp.	#28	2063
or/7-28	#29	42737
exp Mental Disorders/ or mental* disorder*.mp.	#30	925955

Query	Filters	Results
exp "Depression (Emotion)"/ or depres*.mp.	#31	375651
exp major depression/ or depressive* disorder*.mp.	#32	160422
anxiety*.mp. or exp Anxiety/ or exp Anxiety Disorders/	#33	261658
burnout*.mp.	#34	15179
exhaustion*.mp.	#35	7574
exp affective psychosis/	#36	573
Psychotic* Affective* Disorder*.mp.	#37	57
mood* disorder*.mp. or exp Affective Disorders/	#38	158448
exp Antidepressant Drugs/ or antidepressive* agent*.mp.	#39	47066
tricyclic* antidepress*.mp.	#40	4674
antidepressant*.mp.	#41	42777
exp Monoamine Oxidase Inhibitors/ or monoamine* oxidase* inhibitor*.mp.	#42	3536
(serotonin* adj2 noradrenaline* reuptake* inhibitor*).mp.	#43	298
"serotonin and noradrenaline reuptake inhibitor*".mp.	#44	313
psychotropic* drug*.mp.	#45	11420
exp Citalopram/ or citalopram*.mp.	#46	3551
fluvoxamin*.mp.	#47	1775
antimanic*.mp.	#48	2810
exp Distress/ or psychiatric* distress*.mp.	#49	23293
exp Employee Engagement/ or work* engagement*.mp.	#50	4160
exp Mental Health/ or mental* health*.mp.	#51	230540
mental* well*.mp.	#52	3069
mental* ill*.mp.	#53	59983
psychiatric* disorder*.mp.	#54	39516
psychiatric* illness*.mp.	#55	8422
or/30-55	#56	1327761
6 and 29 and 56	#57	3288
limit 57 to english language	#58	3078
**ProQuest EconLit 5.11.2020**		
(workplace* OR flexitime* OR "work* shift*" OR "work* schedule*" OR "flexible* work*" OR flexiwork* OR "flexi* work*" OR "shift* work*" OR "time* management*" OR telecommuting* OR "return* to work*" OR "work* time*" OR "work* life* conciliat*" OR telework* OR "work* hour*" OR "schedule* control*" OR "remote*	English	333

Query	Filters	Results
work*" OR "part-time* work*" OR "work-life* conflict*" OR "work* life* conflict*" OR "work* family*" OR "care* arrangement*" OR "career* break*" OR "job* shar*" OR "term-time* work*" OR "compres* work* time*" OR "self* roster*" OR sabbatic* OR "work* life* reconc*" OR "work-life* reconc*") AND noft((mental* OR depress* OR anxiety* OR burnout* OR "burn-out*" OR exhaustion* OR affective* OR "mood* disorder*" OR antidepres* OR "oxidase* inhibitor*" OR "serotonin* and noradrenalin* reuptake* inhibitor*" OR psychotropic* OR citalopram* OR fluvoxamin* OR antimanic* OR "psychiatric* distress*" OR "work* engagement*" OR "psychiatric* disorder*" OR "psychiatric* illness*")) AND la.exact("English")		
**EconPapers 5.11.2020**<br>workplace* OR flexitime* OR "work* shift*" OR "work* schedule*" OR "flexible* work*" OR flexiwork* OR "flexi* work*" OR "shift* work*" OR "time* management*" OR telecommuting* OR "return* to work*" OR "work* time*" OR "work* life* conciliat*" OR telework* OR "work* hour*" OR "schedule* control*" OR "remote* work*" OR "part-time* work*" OR "work-life* conflict*" OR "work* life* conflict*" OR "work* family*" OR "care* arrangement*" OR "career* break*" OR "job* shar*" OR "term-time* work*" OR "compres* work* time*" OR "self* roster*" OR sabbatic* OR "work* life* reconc*" OR "work-life* reconc*", mental* OR depress* OR anxiety* OR burnout* OR "burn-out*" OR exhaustion* OR affective* OR "mood* disorder*" OR antidepres* OR "oxidase* inhibitor*" OR "serotonin* and noradrenalin* reuptake* inhibitor*" OR psychotropic* OR citalopram* OR fluvoxamin* OR antimanic* OR "psychiatric* distress*" OR "work* engagement*" OR "psychiatric* disorder*" OR "psychiatric* illness*" in titles and keywords.		421

Table S2. Characteristics of studies included in the review.

N of studies	Study (First author and year of publication)	Country	Study design	Follow-up time	Population	Sex distribution	Mean age (or age range) at baseline	Number of participants (included in the analysis)	Intervention or exposure	Outcome	Results	Adjustment for confounders
1	Moen 2016 [18]	USA	Cluster RCT	One year	Employees and managers of a large firm's IT division. The	Among the Early Survey Groups,	Birth year: 1946-1980	865 participants (436 in intervention group	The intervention facilitated working at home, improved employees' control	Burnout, perceived stress, and	Among total sample, the intervention significantly decreased burnout	Gender, age, and managerial status. The results of

N of studies	Study (First author and year of publication)	Country	Study design	Follow-up time	Population	Sex distribution	Mean age (or age range) at baseline	Number of participants (included in the analysis)	Intervention or exposure	Outcome	Results	Adjustment for confounders
					sample consisted of the Early Survey Groups (intervention began before the merger announcement) and the Late Survey Groups (workers completed the baseline survey and received intervention after the merger announcement). In the Early Survey Groups, 16% of control group and 21% of intervention group were managers. Among the Late Survey Groups, 26% of control group and 24% of intervention group were managers.	38% of control group and 36% of intervention group were women. Among the late Survey Groups, 32% of control group and 46% of intervention group were women.		and 429 in control group). 453 in the Early Survey Groups (234 in intervention group and 219 in control group) and 412 in the Early Survey Groups (202 in intervention group and 210 in control group).	over working time, control over shift scheduling, and control over when, where, and how employees do their work. Moreover, it promoted supervisor support for workers' personal lives. The intervention included 8 hours of participatory training sessions.	psychological distress.<br><br>Psychological distress was assessed by a 6-item scale validated by Kessler et al. 2003. Emotional exhaustion was assessed by a 3-item subscale of the Maslach Burnout Inventory. Perceived stress was measured with a 4-item scale (measured only in employees and not in managers).	(P < 0.01). The intervention had an effect only among the Early Survey Groups, but not among the Late Survey Groups. Among the Early Survey Groups, the intervention group had significantly lower levels of burnout (P <0.001), perceived stress (P <0.05), and psychological distress (P <0.05) at 12 months than control group. Cohen's d was 0.36 for burnout, 0.18 for perceived stress, and 0.18 for psychological distress. Changes in schedule control mediated the effect of intervention on psychological distress	unadjusted and adjusted for these variables did not differ.

N of studies	Study (First author and year of publication)	Country	Study design	Follow-up time	Population	Sex distribution	Mean age (or age range) at baseline	Number of participants (included in the analysis)	Intervention or exposure	Outcome	Results	Adjustment for confounders
											by 23%, and on burnout by 19%.	
2	Moen 2011[27]	USA	Non-randomized controlled trial	6 months	White-collar workers of corporate headquarters of Best Buy Co., Inc	48.4% were women	32	659 (325 intervention, 334 control)	An organizational initiative aimed to move employees and supervisors away from conventional practises to environments wherein employees are able to change their work location or schedules and can decide when and where they work based on their own and the team's needs, preferences, and job responsibilities.	Emotional exhaustion and psychological distress.<br><br>Emotional exhaustion was measured with a 5-item subset of items from the Maslach Burnout Inventory.<br><br>Psychological distress was assessed using the 6-item scale of Kessler Psychological Distress Scale (K6).	The natural experiment did not directly lead to changes in emotional exhaustion or psychological distress at follow-up. However, the experiment increased schedule control and decreased negative work-home spillover, which both were associated with lower emotional exhaustion and psychological distress. . There was no significant difference in the associations between men and women.	Gender, age group, parental status, exempt status, income, tenure, occupational level, work hours (more than 50 per week), psychological job demands, decision authority, skill discretion, satisfaction with manager, supportive occupational environment, life change between waves, and job change between waves.

N of studies	Study (First author and year of publication)	Country	Study design	Follow-up time	Population	Sex distribution	Mean age (or age range) at baseline	Number of participants (included in the analysis)	Intervention or exposure	Outcome	Results	Adjustment for confounders
3	Nabe-Nielsen 2011 [28]	Denmark	Non-randomized controlled trial	1 year	Eldercare workers	100% women	Intervention subgroup A 43.9 (10.9), controls 44.7 (10.2)	Intervention subgroup A n=35, controls n=187	Implementation of worktime self-scheduling via a computer program (subgroup A). Each employee planned his/her preferred working hours for 4-6 weeks. The program developed a schedule for the period considering employees' preferences and the workplace's personnel needs.	Self-reported stress.<br><br>It was measured with a Stress-scale and an Energy-scale. Each scale consisted of six items with six response alternatives (0 to 5).	The intervention increased employees' involvement in planning of their working hours (OR=104, 95% CI 11.6-941), but did not decrease stress (mean difference=-0.07, 95% CI -0.40, 0.26 for Stress-scale and -0.08, 95% CI -0.39 to 0.23 for Energy-scale).	Age, job demands, job resources, social support
4	Mache 2020 [16]	Germany	Cross-over study	1 year	Full-time employees of a large technology company headquarter	53.5% were women	39 (9.5)	71	Transition into open workspaces including flexible work arrangements.	Occupational stress and psychological detachment from work.<br><br>Occupational stress was assessed with the 4-item	One year after transition into open workplaces, occupational stress decreased (P value 0.01), but psychological detachment from work did not change.	Age and work experience

N of studies	Study (First author and year of publication)	Country	Study design	Follow-up time	Population	Sex distribution	Mean age (or age range) at baseline	Number of participants (included in the analysis)	Intervention or exposure	Outcome	Results	Adjustment for confounders
										subscale of the COPSOQ II and psychological detachment from work was assessed with the 4-item subscale of the Recovery Experience Questionnaire.	Mean (SD) value for occupational stress was 3.87 (1.05) one month before experiment, 3.61 (1.08) three months after experiment and 3.51 (1.01) one year after experiment. After adjustment for age and work experience, flexible working arrangements was inversely associated with occupational stress (β= −0.17, P< 0.05). There was an interaction between flexible working arrangements and job autonomy (ß = 0.12, P < 0.05) for occupational stress.	

N of studies	Study (First author and year of publication)	Country	Study design	Follow-up time	Population	Sex distribution	Mean age (or age range) at baseline	Number of participants (included in the analysis)	Intervention or exposure	Outcome	Results	Adjustment for confounders
5	Vesala 2015 [17]	Finland	Cross-over study	1-3 months	A sample of knowledge workers including journalists, advertising experts, textile designers, IT developers, well-being coaches, consultants, health care service providers, architects and researchers.	50% were women	44 (11.5)	39	One week telework in the rural archipelago settings mostly in small groups (2-5 persons).	Work engagement, stress, mental exhaustiveness of work.<br><br>Stress and mental exhaustion were assessed with a 5-point Likert-type scale, and work engagement was assessed with a 9-item (0 to 6 points) measure.	The levels of stress were significantly lower during the telework period, and it quite did not reach the original level after the experiment. Mean (SD) value of stress was 2.89 (0.98) before teleworking, 2.55 (0.80) during teleworking and 2.68 (0.93) after teleworking.<br><br>Telework had no significant effect on work engagement. Mental exhaustiveness decreased during intervention, but it returned to the original level after experiment.	None

N of studies	Study (First author and year of publication)	Country	Study design	Follow-up time	Population	Sex distribution	Mean age (or age range) at baseline	Number of participants (included in the analysis)	Intervention or exposure	Outcome	Results	Adjustment for confounders
6	Albrecht 2020a [29]	Finland	Prospective cohort study	7 years	Full-time public sector employees	75% were women	39% were 50 years or older	22599	Worktime control was assessed using 7-item measure, but control over daily hours (length of a workday, starting and ending times) and control over time off (scheduling of vacation and unpaid leave) were studied.	Sickness absence for 10 days or longer due to depression or anxiety	Both control over daily hours and control over time off were not associated with sickness absence due to depression or anxiety.<br><br>Adjusted OR was 1.08 (95% CI 0.97-1.21) for moderate and 1.06 (CI 0.95-1.19) for high control over daily hours compared with low control.<br><br>Adjusted OR was 1.05 (95% CI 0.95-1.17) for moderate and 0.95 (CI 0.86-1.06) for high control over time off compared with low control.	Sex, age, occupational status, educational attainment, shift work, and physical workload.

N of studies	Study (First author and year of publication)	Country	Study design	Follow-up time	Population	Sex distribution	Mean age (or age range) at baseline	Number of participants (included in the analysis)	Intervention or exposure	Outcome	Results	Adjustment for confounders
6	Vahtera 2010 [30]	Finland	Prospective cohort study	4-5 years (mean 4.4)	Public sector employees	77.5% were women	44.8 (9.3)	30700	Self-assessed and co-worker assessed worktime control. It was assessed by the 7-itemmeasure.	Disability pension due to mental disorders	Self-assessed, but not co-worker assessed worktime control was associated with lower risk of disability pension due to mental disorders among women.<br><br>For 1-unit increase in self-assessed worktime control, hazard ratio was 0.79 (95% CI 0.68-0.92) for women, 0.89 (95% CI 0.66-1.19) for men and 0.81 (0.71-0.93) for men and women combined.	Age and socioeconomic status
6	Ala-Mursula 2004 [31]	Finland	Prospective cohort study	3 years	Permanent full-time municipal employees	78.6% were women	Mean age at baseline was 45.7 (SD 7.7) years for men	4218	Worktime control was assessed by 6 items. Worktime control was defined as high for participants with	Psychological distress was measured by the 12 -item Goldberg's General	OR of psychological distress was 1.40 (95% CI 1.08 - 1.82) for women with a low level of worktime control	Age, occupational status, marital status, dependent children, baseline level of

N of studies	Study (First author and year of publication)	Country	Study design	Follow-up time	Population	Sex distribution	Mean age (or age range) at baseline	Number of participants (included in the analysis)	Intervention or exposure	Outcome	Results	Adjustment for confounders
							and 44.7 (SD 7.4) for women.		highest tertile in both 1997 and 2000, defined as low for participants with the lowest tertile for both years, and defined as intermediate for all other combinations.	Health Questionnaire (GHQ-12).	compared with women with a high level of worktime control. Worktime control was not associated with psychological distress among men (OR 0.92, 95% CI 0.57 to 1.49).<br><br>Among participants not psychologically distressed at baseline, OR of psychological distress was 1.91 (95% CI 1.22 - 3.01) for women with a low level of worktime control compared with women with a high level of worktime control, and was 1.27 (95% CI 0.49 - 3.32) for men with a low level of worktime control	psychological distress, smoking, alcohol consumption, overweight, and sedentary lifestyle.

N of studies	Study (First author and year of publication)	Country	Study design	Follow-up time	Population	Sex distribution	Mean age (or age range) at baseline	Number of participants (included in the analysis)	Intervention or exposure	Outcome	Results	Adjustment for confounders
											compared with men with a high level of worktime control.	
7	Aronsson 2019 [32]	Sweden	Prospective cohort study	2 years	General working population who worked at least 30% full-time	58 % women	51 (10)	4408	Worktime control.<br><br>It appears that several items were used to measure worktime control. Low work-time control was defined as lowest quartile.	Burnout.<br><br>It was measured using the 8-item of the Shirom-Melamed Burnout Questionnaire	Population attributable fraction of burnout for low worktime control was 5% for human service occupations and 7% for other occupations.	Age, gender, civil status, education, organizational resources (insufficient human resources and insufficient economic resources) and other psychosocial work factors including high rewards, high efforts, low social support, high organizational injustice, high emotional demands and

N of studies	Study (First author and year of publication)	Country	Study design	Follow-up time	Population	Sex distribution	Mean age (or age range) at baseline	Number of participants (included in the analysis)	Intervention or exposure	Outcome	Results	Adjustment for confounders
												violence or threat of violence
8	Lee 2018 [33]	USA	Prospective cohort study, 3 follow-up time points (6, 12 and 18 months)	1.5 years	Employees from 26 different technology offices. This study was originally conducted as a randomized controlled trial, however, in the current study only the control group was included in the analysis.	Not reported. In the original study (randomized controlled trial), 45% were women.	Not reported	507	Control over working hours/schedule assessed by an 8-item developed by Thomas and Ganster in 1995.	Burnout was assessed using the Maslach Burnout Inventory. Emotional exhaustion, a subscale of the Maslach Burnout Inventory was also used. It includes three items. Burnout was measured at 4 time points.	Control over working hours/schedule at baseline was statistically significantly correlated with burnout at baseline (- 0.30), 6 months follow-up (- 0.26), 12 months (-0.24) and 18 months (-0.24). Control over working hours/schedule had no statistically significant effect on initial status of burnout (β = - 0.07). Control over working hours/schedule had also no statistically significant effect on the slope of burnout (β = 0.03).	In addition to unconditional growth model, a conditional growth model was also run, and background characteristics were included in the model.

N of studies	Study (First author and year of publication)	Country	Study design	Follow-up time	Population	Sex distribution	Mean age (or age range) at baseline	Number of participants (included in the analysis)	Intervention or exposure	Outcome	Results	Adjustment for confounders
9	Albrecht 2017 [34]	Sweden	Prospective cohort study	6 years	A population-based sample of working people	58.6% were women	47.0 (8.3)	2722	Worktime control was assessed by 5 items. However, only two items were used in the analysis (control over daily hours, control over time off).	Depressive symptoms were assessed using the 6-item subscale of the Hopkins Symptom Checklist (SCL-90). Depression was defined as score 17 or higher.	Low control over daily hours and low control over time off were significantly associated with higher subsequent depressive symptoms. There was no gender difference.	Age, sex, education, weekly working hours, occupation and shift work.
9	Albrecht 2020b [35]	Sweden	Prospective cohort study	6 years	A population-based sample of working people	55.4% were women	49 (11.8)	26804	Worktime control was assessed by 5 items. However, only two items were used in the analysis. (control over daily hours, control over time off).	Depressive symptoms were assessed using the 6-item subscale of the Hopkins Symptom Checklist (SCL-90). Depression was defined as score 17 or higher.	Work-life imbalance partially mediated the relationship between worktime control and depressive symptoms. Indirect effects were small, but the estimate for control over time off was in general larger than for control over daily hours.	Sex, age, education, socioeconomic status, shift work, weekly working hours, civil status, and parental status

N of studies	Study (First author and year of publication)	Country	Study design	Follow-up time	Population	Sex distribution	Mean age (or age range) at baseline	Number of participants (included in the analysis)	Intervention or exposure	Outcome	Results	Adjustment for confounders
10	Windeler 2017 [36]	USA	A prospective cohort study	4 months	Employees of the IT business unit of a financial services firm	39% were women	Mean age was 43 years	51	Participants teleworked for one or two days per week over 4 months. More than half (54%) teleworked two days per week.	Change in work exhaustion. Work exhaustion was measured a week before part-time telework and at 4 months using a four-item scale from the Maslach Burnout Inventory.	Work exhaustion increased as interpersonal interaction increased. Part-time telework reduced the effect of interpersonal interaction on work exhaustion. The coefficient for positive association between interpersonal interaction and work exhaustion decreased from 0.28 in baseline survey to 0.03 in follow-up survey. External interaction was not statistically significantly associated with work exhaustion. Part-time telework potentiates the effect of external interaction on work exhaustion. The coefficient for the	Age and sex

N of studies	Study (First author and year of publication)	Country	Study design	Follow-up time	Population	Sex distribution	Mean age (or age range) at baseline	Number of participants (included in the analysis)	Intervention or exposure	Outcome	Results	Adjustment for confounders
											association between external interaction and work exhaustion increased from -0.11 in baseline survey to 0.37 (30% of the variance in work exhaustion) in follow-up survey. There was no interaction between Interdependence and telework on work exhaustion.	
11	Henke 2016 [37]	USA	Prospective cohort study	2 years?	Active Prudential Financial employees who had continuous medical enrolment between 2010 and 2011 and had completed health risk assessment in 2010 and 2011.	62% were women.	18 to 64 years. 88% were younger than 55 years.	3703	Prime time telecommuters who worked 51% or more of their remote hours during prime work hours. Off-hour telecommuters who worked 50% or less of their remote hours during prime work hours.	Depression: participants were asked whether they had felt down, depressed, or hopeless during the past 2 weeks. Stress: participants were asked whether	There was a U-shaped or J-shaped association between the number of hours worked from home per month and depression. Non-telecommuters were at higher risk of depression than telecommuters.	Age, sex, job grade, ethnicity, management status, region, and a diagnosis cost grouper.

N of studies	Study (First author and year of publication)	Country	Study design	Follow-up time	Population	Sex distribution	Mean age (or age range) at baseline	Number of participants (included in the analysis)	Intervention or exposure	Outcome	Results	Adjustment for confounders
									Prime time telecommuting was grouped into <=8, 9 to 32, 33 to 72, and >=73 hours per month.	in the past year, stress has affected their health or well-being.	Telecommuter who worked from home for <=8 hours per month reduced their risk for depression more than non-telecommuters during follow-up period (β = −0.276, $p < 0.05$). The difference was not statistically significant for off-hour telecommuting and prime time telecommuting for 9 to 32, 33 to 72, and >=73 hours per month. The results were not also statistically significant for stress.	
12	Kubo 2016 [38]	Japan	Prospective cohort study	1 year	Employees of a manufacturing industry and a research institute	23% were women	Mean age 41.9 years (SD 12.8)	37	Worktime control was assessed by 5 items: 1) length of workday, 2) starting and ending	Accumulated fatigue'' was assessed using 13 items. The questionnaire	Increase in worktime control during 1-year follow-up was not associated	Age, sex, and job position.

N of studies	Study (First author and year of publication)	Country	Study design	Follow-up time	Population	Sex distribution	Mean age (or age range) at baseline	Number of participants (included in the analysis)	Intervention or exposure	Outcome	Results	Adjustment for confounders
									times of workday, 3) number of breaks during workday, 4) vacations and paid days off, and 5) unpaid leave.	included items on irritability, anxiety, restlessness, depressed mood, lack of concentration, and lack of motivation in the past month.<br><br>Fatigue was also objectively measured by a psychomotor vigilance task, a sustained-attention reaction time task with a random inter-stimulus interval of 2 to 10 seconds.	with accumulated fatigue.<br><br>There was an association between increase in worktime control during 1-year follow-up and objectively measured fatigue. The number of lapses on the second half of the workday, measured by a psychomotor vigilance task, at 1-year follow-up was statistically significantly lower in workers who increased their worktime control during 1-year follow-up than in workers who did not (P <0.05). A similar result was found for speed, but	

N of studies	Study (First author and year of publication)	Country	Study design	Follow-up time	Population	Sex distribution	Mean age (or age range) at baseline	Number of participants (included in the analysis)	Intervention or exposure	Outcome	Results	Adjustment for confounders
											difference was borderline significant (P <0.10).	
13	Shepherd-Banigan 2016 [39]	USA	Prospective cohort study, 3 follow-up time points (6, 15 and 24 months)	2 years	Mother new-born dyads were recruited from 24 hospitals within 10 US cities. Working women who returned to work within 6 months after childbirth and worked at 6, 15, and 24 months after birth.	All participants were women	Age ranged between 18 and 43. Mean age was 29.6 (4.7) years.	570	Schedule flexibility and working from home. Schedule flexibility was based on one item: how flexible are your work hours? Works from home compared with no work from home. Number of hours worked from home was studied among 132 participants.	Depressive symptoms measured by the Center for Epidemiologic Studies Depression Scale at 1, 6, 15, and 24 months.	Schedule flexibility and number of hours worked from home were not associated with changes in depressive symptoms. B (SE) was 0.23 (0.98) for very flexible schedule, 0.19 (0.92) for fairly flexible schedule and 0.18 (0.89) for minimally flexible schedule compared with not flexible schedule. Working from home statistically significantly reduced depressive symptoms. B (SE) was −1.36 (0.51) (p <0.05) for working at home compared	The estimates for schedule flexibility controlled for work intensity and work from home. The estimate for work from home controlled for work intensity. It is unclear whether the study adjusted for other factors.

N of studies	Study (First author and year of publication)	Country	Study design	Follow-up time	Population	Sex distribution	Mean age (or age range) at baseline	Number of participants (included in the analysis)	Intervention or exposure	Outcome	Results	Adjustment for confounders
											with not working at home.	
14	Timms 2015 [40]	Australia	Prospective cohort study	1 year	Employees from 8 organisations in eastern states of Australia representing education, banking, and public and community services	72% were women	43 (10.3)	823	Flexible work arrangements included 4 items: 1) flexitime, 2) compressed working week, 3) telecommuting, and 4) part-time work)	Psychological strain, work engagement.<br><br>The 4-item from the General Health Questionnaire was used to assess psychological strain.<br><br>Work engagement was measured with the 9-item short version of the Utrecht work engagement Scale.	Use of flexible work arrangement at baseline was inversely associated with work engagement at follow-up (β -0.17, P <0.001). Using flexible work arrangements was positively associated with psychological strain (β 0.08, P <0.05).	Age, gender, civil status (being married and/or having children), supervisor support, organizational support, organizational time demands, and negative career consequences
15	Takahashi 2012 [41]	Japan	Prospective	15-16 months	Daytime managers, professionals, clerical workers,	34.8% were women.	20 to 59 years, mean age	2382	Worktime control was assessed by 5 items. The change	Fatigue was measured with 11 items	Low–high and high–high groups had lower levels of	Age, gender, occupation, weekly and

N of studies	Study (First author and year of publication)	Country	Study design	Follow-up time	Population	Sex distribution	Mean age (or age range) at baseline	Number of participants (included in the analysis)	Intervention or exposure	Outcome	Results	Adjustment for confounders
			cohort study		sales workers, or transportation workers. Approximately 20% were managers.		40.6 ± 10.5 years		in work time control between baseline and follow-up was classified into four groups: low to low, low to high, high to low, and high to high.	of the Checklist for Accumulated Fatigue due to Overwork. Depressive symptoms were measured by the Center for Epidemiological Studies Depression Scale. Recovery from fatigue was assessed using a single question.	fatigue and depressive symptoms at follow-up than low–low and high–low groups. Recovery from fatigue was significantly higher in participants who their work time control was high at both baseline and follow-up than in those with low at both baseline and follow-up.	psychosocial work characteristics.
16	Hornung 2011 [42]	Germany	Prospective cohort study	1 year	Medical doctors from 2 German hospitals	46.5% were women	39.4 (9.0)	91	Flexibility idiosyncratic deals, which were measured with 3 items: 1) working time flexibility, 2) work schedule flexibility, and 3) influence over working hours.	Work-family conflict, work-related well-being (work engagement)<br><br>The 5-item scale was used to assess	Cross sectionally, flexibility idiosyncratic deals were inversely associated with work-family conflict but there was no association between flexibility idiosyncratic deals	Cross sectional associations were controlled for age, sex, part-time employment, job tenure, and occupational position.

N of studies	Study (First author and year of publication)	Country	Study design	Follow-up time	Population	Sex distribution	Mean age (or age range) at baseline	Number of participants (included in the analysis)	Intervention or exposure	Outcome	Results	Adjustment for confounders
										work-family conflict.	and work engagement.	
										The German version of the Utrecht Work Engagement Scale was used to assess work-related well-being (work engagement).	Longitudinally, idiosyncratic deals were neither associated with work-family conflict nor with work engagement.	

Table S3. Methodological quality of the included randomized controlled trial.

Bias domain	Moen 2016
Bias arising from the randomization process	Some concerns
Bias arising from the timing of identification and recruitment of individual participants in relation to timing of randomization	Low
Bias due to deviations from intended interventions	Low
Bias due to missing outcome data	Some concerns
Bias in measurement of the outcome	High
Bias in selection of the reported result	Low
Overall bias	High

Table S4: Methodological quality of the included non-randomized controlled trials.

Bias domain	Moen 2011, 2013	Nabe-Nielsen 2011
Bias due to confounding	Moderate	Moderate
Bias in selection of participants into the study	Moderate	Moderate
Bias in classification of interventions	Low	Low
Bias due to deviations from intended interventions	Low	Low
Bias due to missing data	Moderate	Moderate
Bias in measurement of outcomes	Low	Low
Bias in selection of the reported result	Low	Low
Overall bias	Moderate	Moderate

Table S5. Methodological quality of the included cross-over studies.

Bias domain	Vesala 2015	Mache 2020
Appropriate cross-over design	High	High
Randomized treatment order	High	High
Carry-over effect	Unclear	Unclear
Unbiased data	Low	Low
Allocation concealment	High	High
Blinding	High	High
Incomplete outcome data	Unclear	Unclear
Selective outcome reporting	Low	Low
Other bias	Unclear	Unclear

Table S6. Methodological quality of the included prospective cohort studies using the JBI Critical Appraisal Checklist for Cohort Studies.

Quality checklist	Albrecht 2020a	Vahtera 2020	Ala-Mursula 2004	Aronsson 2019	Lee 2018	Albrect 2017	Al-brecht 2020b	Windeler 2017	Henke 2016	Kubo 2016	Shep-herd-Ban-igan 2016	Timms 2015	Takahashi 2012	Hor-nung 2011
1. Were the two groups similar and recruited from the same population?	Y	Y	Y	Y	Y	Y	Y	Y	Y	U	Y	Y	Y	Y
2. Were the exposures measured similarly to assign people to both exposed and unexposed groups?	Y	Y	Y	Y	Y	Y	Y	Y	Y	Y	Y	Y	Y	Y
3. Was the exposure measured in a valid and reliable way?	Y	Y	Y	Y	Y	Y	Y	Y	Y	Y	?	Y	Y	Y
4. Were confounding factors identified?	Y	N	Y	Y	U	Y	Y	N	Y	Y	U	Y	Y	Y
5. Were strategies to deal with confounding factors stated?	Y	N	Y	Y	U	Y	Y	U	Y	Y	U	Y	Y	Y
6. Were the groups or participants free of the outcome at the start of the study (or at the moment of exposure)?	Y	Y	Y	N	N	N	N	N	N	N	N	N	N	N
7. Were the outcomes measured in a valid and reliable way?	Y	Y	Y	Y	Y	Y	Y	Y	N	Y	Y	Y	Y	Y
8. Was the follow up time reported and sufficient to be long enough for outcomes to occur?	Y	Y	?	Y	Y	Y	Y	U	Y	Y	Y	Y	?	Y
9. Was follow up complete, and if not, were the reasons	Y	Y	Y	N	N	N	N	Y	N	Y	N	N	N	N

Quality checklist	Albrecht 2020a	Vahtera 2020	Ala-Mursula 2004	Aronsson 2019	Lee 2018	Albrect 2017	Al-brecht 2020b	Windeler 2017	Henke 2016	Kubo 2016	Shep-herd-Ban-igan 2016	Timms 2015	Takahashi 2012	Hor-nung 2011
to loss to follow up described and explored?														
10. Were strategies to address incomplete follow up utilized?	NA	NA	N	N	Y	N	N	NA	N	NA	Y	N	N	N
11. Was appropriate statistical analysis used?	Y	Y	Y	Y	Y	Y	Y	Y	Y	Y	Y	Y	Y	Y
**Total of "yes" scores**	**10**	**8**	**9**	**8**	**7**	**8**	**8**	**6**	**7**	**8**	**6**	**8**	**7**	**8**

Y, yes; N, no; U, unclear; NA, not applicable.
